# Proof-of-principle: targeted childhood leukemia prevention

**DOI:** 10.18632/oncotarget.28371

**Published:** 2023-03-11

**Authors:** César Cobaleda, Manuel Ramírez-Orellana, Carolina Vicente-Dueñas, Andreas Weiss, Kim E. Nichols, Isidro Sánchez-García

**Keywords:** leukemia, infection, murine models, genetic susceptibility, prevention

Cancer is the most common cause of disease-related childhood mortality in developed countries, with B-cell acute lymphoblastic leukemia (B-ALL) representing the most frequent. A salient characteristic underlying some cases of childhood B-ALL is the presence of congenital mutations (either inherited or *de novo*) that are compatible with normal lymphocyte development, but lead to the appearance of a silent population of preleukemic cells that acquire additional genetic mutations and ultimately progress to full-blown B-ALL. The precise mechanisms underlying malignant transformation have been difficult to ascertain due to their development in otherwise healthy-appearing children. Notably, several decades ago, delayed exposure to common infections was postulated as a triggering factor leading to the appearance of the secondary genetic lesions required for B-ALL to develop [1].

Using mouse models genetically engineered to carry leukemia-predisposing germline mutations found in human B-ALL patients, several recent studies have demonstrated the existence of this infection-triggered process in the progression towards B-ALL, providing evidence that different types of immune stress can activate the clonal evolution of preleukemic precursors [2–4]. One key aspect identified through these studies is that the transforming effect of infection does not result from the outgrowth of preleukemic clones already carrying one or more second hits; instead, the effect of infection-induced immune stress is to trigger the actual appearance of these second mutations, thereby directly causing progression to B-ALL [1]. Under these premises, it becomes conceivable that one could prevent the development of B-ALL by eliminating the preleukemic clone [1–5]. However, one would have to find a way to specifically target these preleukemic cells. Recently, a mouse model recapitulating the phenotype of a leukemia-predisposition syndrome has allowed us to carry out a proof-of-principle experiment to achieve this very goal.

Children carrying heterozygous mutations affecting the B-cell master regulator gene *PAX5* are predisposed to develop B-ALL; similarly, 25% of heterozygous *Pax5*^+/−^ mice develop leukemia, but only after experiencing an immune stress, such as exposure to infection [2–4]. Furthermore, the B-leukemias that appear in *Pax5*^+/−^ animals acquire similar mutations as observed in the leukemic blasts from humans harboring pathogenic *PAX5* variants, including activating mutations affecting the Janus Kinases (JAKs) [2]. We previously demonstrated that, in *Pax5*^+/−^ mice, early B cell precursors (pro-B cells) are very dependent upon interleukin-7 (IL-7) for their survival. Further, blockade of IL-7 signaling by treatment with the JAK1/2 inhibitor ruxolitinib led to apoptosis of *Pax5*^+/−^ pre-leukemic B cells *in vitro* [2]. Taking advantage of this knowledge, we have recently used *Pax5*^+/−^ mice to evaluate whether *in vivo* treatment with ruxolitinib early in life will kill preleukemic cells and, therefore, prevent the development of acute leukemia [6]. Pharmacokinetic studies were performed to determine appropriate doses and then wild-type (WT) and *Pax5*^+/−^ mice were fed with ruxolitinib in their chow. Mirroring the *in vitro* results indicating high dependence on IL-7 signaling, treatment with ruxolitinib mainly led to the disappearance of B-cell progenitors in *Pax5*^+/−^, but not in WT, animals [6].

**Figure 1:**
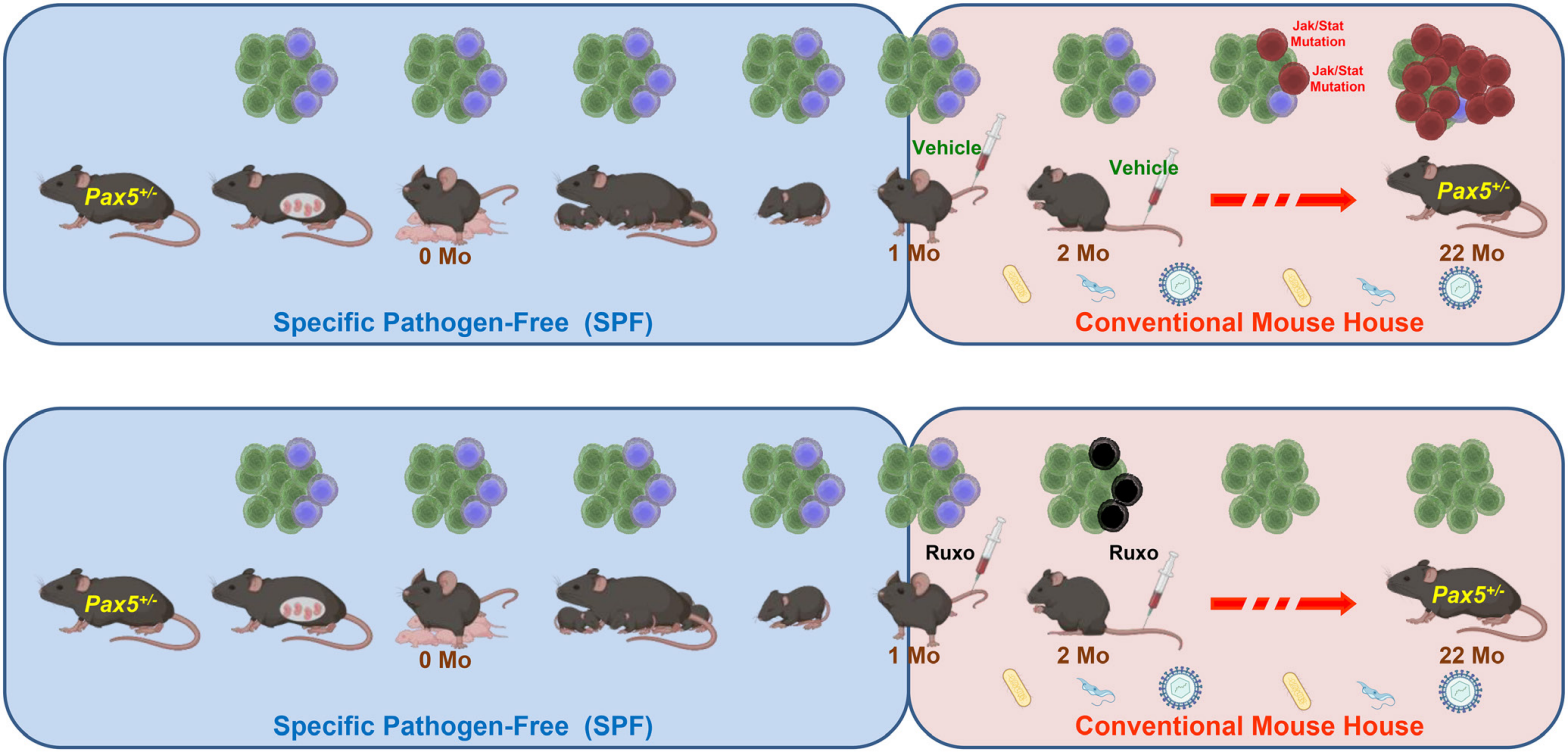
Targeted prevention of progression to B-ALL. In most *Pax5*^+/−^ mice, preleukemic progenitor B cells (shown as blue cells) are compatible with a normal hematopoietic development. However, immune stressors, such as exposure to common mouse pathogens after transfer to a non-SPF animal facility, trigger progression to B-ALL (red cells) through the appearance of secondary mutations affecting the Jak/Stat pathway. Transient treatment of *Pax5*^+/−^ mice with the Jak1/2 inhibitor ruxolitinib destroys these progression-prone preleukemic cells and significantly reduces the risk of leukemia development. These results demonstrate that there is a window of opportunity in early postnatal life during which preventing the progression to B-ALL in predisposed children might be possible. Mo: months-old.

Therefore, in the next experiment, both experimental *Pax5*^+/−^ and control WT animals were fed with ruxolitinib containing chow for 14 or 28 days, starting from the moment they were transferred from a specific-pathogen-free animal house (SPF, where they had been born and weaned) to a conventional facility, where they were exposed to common mouse pathogens (Figure 1). The animals treated with ruxolitinib for the longer period (28 days) exhibited a significant 90% reduction in the incidence of B-ALL when compared to untreated mice, or to animals treated only for 14 days [6].

Ultra-deep sequencing studies of *Pax5*^+/−^ mice had previously shown that the activating mutations affecting *Jak* are only detected once the animals already present with the full-blown B-ALL, and never during the preleukemic period [2]. Furthermore, when a constitutively activated *Jak3^V670A^* transgene is added to the *Pax5*^+/−^ genotype, the animals develop B-ALL immediately and without the need of exposure to any immune stress. Therefore, it is unlikely that the reduction of the incidence of acute leukemia after treatment with a Jak inhibitor for 28 days is due to the selective death of *Pax5*^+/−^ progenitors already harboring leukemogenic mutations in this pathway. On the contrary, one can conclude that ruxolitinib is acting by eliminating predisposed preleukemic B cells before the second hit leading to B-ALL arises in them (Figure 1).

These data show that ruxolitinib-mediated inhibition of the JAK-STAT pathway in predisposed *Pax5*^+/−^ animals prevents the development of leukemia, and suggest that an analogous approach could be used to prevent progression to B-ALL in children at increased genetic risk, either because they are carriers of *PAX5* or due to other predisposing germline mutations. Importantly, since the beneficial action of ruxolitinib in predisposed animals could be achieved following transient treatment with the drug, one can extrapolate that any future treatment of predisposed children need not be sustained for a long period, but just administered during a specific time window during their childhood.

It is becoming increasingly clear that the existence of latent pretumoral cells is common to many types of both hematologic and solid cancers [7]; therefore, the concept described here could be considered a proof-of-principle strategy for the development of similar prophylactic approaches to prevent the progression of other malignancies. Still, some aspects remain unclear. For example, why do the majority of genetically predisposed animals (and most genetically predisposed children) not develop leukemia and stay healthy? In addition, what are the mechanisms by which environmental factors such as infection promote the acquisition of secondary mutations leading to malignant progression of preleukemic cells? These and other important questions still need to be answered if we are to fully understand and avert the appearance of B-ALL.
